# Mechanisms of Cannabidiol (CBD) in Cancer Treatment: A Review

**DOI:** 10.3390/biology11060817

**Published:** 2022-05-26

**Authors:** Camren G. Heider, Sasha A. Itenberg, Jiajia Rao, Hang Ma, Xian Wu

**Affiliations:** 1 Department of Kinesiology, Nutrition, and Health, Miami University, Oxford, OH 45056, USA; heidercg@miamioh.edu (C.G.H.); itenbeaa@miamioh.edu (S.A.I.); 2 Department of Plant Sciences, North Dakota State University, Fargo, ND 58102, USA; jiajia.rao@ndsu.edu; 3 Department of Biomedical and Pharmaceutical Sciences, College of Pharmacy, University of Rhode Island, Kingston, RI 02881, USA

**Keywords:** cannabidiol (CBD), cannabinoid, *Cannabis*, cancer, apoptosis

## Abstract

**Simple Summary:**

Emerging evidence suggests positive outcomes from the use of CBD as a cancer treatment. CBD can relieve cancer pain and ease the side effects of chemotherapy; however, there is less research about the mechanism of CBD’s anticancer effects. In this article, recent studies on the efficacy and mechanisms of CBD’s anticancer effects in cell- and animal-based models and human clinical studies are reviewed.

**Abstract:**

*Cannabis sativa* L. (*Cannabis*) and its bioactive compounds, including cannabinoids and non-cannabinoids, have been extensively studied for their biological effects in recent decades. Cannabidiol (CBD), a major non-intoxicating cannabinoid in *Cannabis*, has emerged as a promising intervention for cancer research. The purpose of this review is to provide insights into the relationship between CBD and cancer based on recent research findings. The anticancer effects of CBD are mainly mediated via its interaction with the endocannabinoid system, resulting in the alleviation of pain and the promotion of immune regulation. Published reviews have focused on the applications of CBD in cancer pain management and the possible toxicological effects of its excessive consumption. In this review, we aim to summarize the mechanisms of action underlying the anticancer activities of CBD against several common cancers. Studies on the efficacy and mechanisms of CBD on cancer prevention and intervention in experimental models (i.e., cell culture- and animal-based assays) and human clinical studies are included in this review.

## 1. Introduction

Phytochemicals, including phytocannabinoids and non-phytocannabinoids, from *Cannabis sativa* L. (*Cannabis*) have been investigated in many published studies in recent years, especially since the legalization of *Cannabis* in many countries and regions. The chemotypes of *Cannabis* can be classified by the levels of their two major phytocannabinoids, namely, ∆9-tetrahydrocannabinol (THC, potentially intoxicating) and cannabidiol (CBD, non-intoxicating). *Cannabis* sp. with low THC content (<0.4%) but high CBD levels is known as hemp or industrial hemp, and is generally grown for industrial purposes (e.g., textiles, rope, clothing, biofuel, and animal feed). *Cannabis* sp. with high THC content is cultivated for medical applications and/or recreational use [1]. *Cannabis* plants are easy to cultivate, as they do not require a specific type of soil; however, the soil should be at a neutral pH or slightly alkaline [1]. Nitrogen is the main component in the soil that interacts with *Cannabis* plants and contributes their THC content [1]. *Cannabis* sp. has evolved as a potential energy crop, which is a crop grown for use in the generation of energy or the production of fuels. For example, bioethanol cannabinoids have high biomass content, high land use efficiency, low nutrient requirements, and no pesticide demand, and they can improve the health of soil with organic matter. They can also be effectively grown in diverse climates and can be used in organic crop rotation [2]. Moreover, phytocannabinoids are extracted from *Cannabis* plants and used to obtain various potential health benefits—including cancer treatment and pain management [3].

CBD is a major bioactive, non-intoxicating cannabinoid in *Cannabis* plants. Many *Cannabis* strains contain higher amounts of CBD than THC [4]. Emerging evidence suggests positive outcomes when using CBD as a cancer treatment. One of the major mechanisms of CBD’s anticancer effects is attributed to its interactions with the endocannabinoid system (ECS), resulting in the alleviation of pain as well as the promotion of immune-cell regulation [3]. The best known mechanism of action of cannabinoids (i.e., CBD) is the mediation of endocannabinoid receptors. Cannabinoids may exert their anticancer effects, as well as many of their other biological activities, primarily by binding to a group of G protein-coupled receptors, known as type 1 and 2 cannabinoid receptors (CB1 and CB2, respectively) [5]. CB1 and CB2 receptors can both exert anti-inflammatory, pro-apoptotic, and antiproliferative effects, which may help fight against cancer [6]. The differences between these receptors are due to where they are distributed in the body [6]. CB1 receptors are found mainly in relation to the nervous system, at the ends of axons, where they are able to inhibit neurotransmitter action and improve with management of pain [6]. CB2 receptors, on the other hand, are highly expressed in peripheral tissues and related to immune functions. CB2 receptors are involved in the control and regulation of cytokinesis in the immune cells, ultimately helping with anticancer factors relating to cell division, such as antiproliferation [6]. CB1 receptors have also been reported to regulate various biological functions, such as appetite, metabolism, and body weight [7], which can be helpful when they are used as treatments for chemotherapy-associated symptoms. Moreover, the ECS is strongly involved in neuropsychiatric symptoms and disorders, such as anxiety and depression [8], which are frequent problems for cancer patients [9]. THC exerts psychotropic effects due to its high affinity with cannabinoid receptor (CB1) in the brain, whereas non-psychotropic cannabinoids, such as CBD, have a lower affinity with CB1, which can be activated to promote the positive effects of CBD [10]. In fact, CBD has a lower affinity with both CB1 and CB2 receptors compared to THC. The anti-proliferative effects of CBD and CBD-enriched extracts against cancer cells are associated with their pro-apoptotic effects and their ability to activate the CB2 receptor [11]. CBD behaves as an agonist of transient potential vanilloid (TRPV) receptors (type 1 and type 2) with no noxious effects [11,12]. TRPV1 and TRPV2 channels are involved in nociception and thermosensing; however, they are aberrantly expressed in several tumor types [13]. Emerging evidence suggests that the activation of TRPV1 by a natural agonist (i.e., capsaicin) contributes to its anticancer effects [14].

One way in which cannabinoids have been clinically used in cancer treatment is through an oromucosal spray containing THC and CBD. This spray was used in cancer patients with severe pain that was not resolved by applying typical opioid therapy [15]. Another study found that the pain relief offered by THC and CBD oromucosal spray lasted over time, suggesting that no tolerance was built and that the dose did not need to be increased after a period of time [16]. A more effective cannabinoid-based pain reliever for cancer-related pain was found to be a THC:CBD combination, which was more efficacious compared to THC alone [11]. When gauging patients’ acceptance of CBD treatments for chemotherapy side effect reduction, it was found that the majority of cancer patients preferred THC:CBD over placebo and that no serious side effects or events occurred as a result of the THC:CBD co-treatment [11]. Published reviews have focused on the application of cannabinoids in cancer pain management and their possible toxicological effects with excessive consumption. Common side effects include somnolence, nausea, dizziness, dry mouth, disorientation, euphoria, anxiety, and hallucination, as well as some more severe adverse events, such as memory or cognitive problems, addiction, and exacerbation or the provocation of mental disorders and pre-existing heart disease [17]. It should be noted that the side effects associated with cannabinoids or *Cannabis* vary from person to person (i.e., genetics, environmental factors, pre-existing conditions, etc.) and are largely dependent on the drug dosage/frequency and cannabinoid constituents [17]. Specifically, toxicological effects are mostly associated with the high dose consumption of THC, whereas CBD is thought to alleviate some of these adverse events. For example, CBD is found to facilitate learning and ameliorate psychosis and anxiety [18]. Thus, in the current review, our particular interest is in CBD as a non-intoxicating cannabinoid. In this review, we sought to summarize the detailed mechanisms of action underlying the anticancer activities of CBD on the four most common cancer types, i.e., lung, breast, prostate, and colorectal cancer [19]. Recent studies that include relevant information about the efficacy and mechanisms of action of CBD on cancer in cell culture and animal studies are reviewed in this article.

## 2. General Characteristics of CBD in Cancer

The non-intoxicating nature of CBD is of importance because it allows greater patient acceptance as a form of cancer treatment. Non-intoxicating cannabinoids, including CBD, which are generally associated with less dramatic, non-intoxicating side effects, have been used more frequently in cancer management in recent years [20]. CBD is generally well tolerated and has a favorable safety profile [21]. The known beneficial effects of CBD on cancer include tumor cell repression, the relief of cancer-related pain, and the reduction of chemotherapy effects, such as nausea and vomiting [22]. Various cannabinoids have been found to attenuate cell proliferation in different cancer cell lines. Despite their structural similarity, cannabinoids may have different pharmacological effects and contributions. In a study that compared the inhibitory effects of CBD, cannabichromene (CBC), cannabigerol (CBG), and tetrahydrocannabivarin on cancer cells’ growth, it was found that CBD had the greatest anti-proliferative effects against prostate cancer cells [23].

Apart from having effects against cancer cells, CBD in combination with THC has been shown to be a promising treatment for cancer pain. For example, a clinical study suggested that a THC:CBD combination was an efficient treatment for pain relief in cancer patients compared to THC alone [16]. Patients also responded to novel combinations of cannabinoids when opioid therapy treatment was insufficient [24]. In a follow-up study of a three-arm clinical trial, patients were able to self-titrate nabiximols, a CBD:THC combination oral mucosal spray, to their preferred level for analgesia and comfort. In addition, lower levels of cancer-related pain, as well as of insomnia and fatigue, were reported [24]. Notably, these cancer patients were able to stay at the same or similar dose without increasing the dosage, and the analgesic effect of the spray treatment remained at the same level [24]. Overall, these clinical studies showed positive outcomes of the long-term use of cannabinoids as analgesic treatments for cancer-related pain. Cannabinoids can also be used to reduce the adverse effects associated with chemotherapy. When compared to a placebo for reducing chemotherapy-induced nausea and vomiting, a CBD:THC combination showed significant improvement. Some side effects occurred along with the cannabinoids, such as dizziness and disorientation, but there were no severe adverse effects and the treatment was tolerated well [12].

Although the efficacy of CBD or co-treatment with CBD and other cannabinoids in alleviating cancer pain and the side effects associated with chemotherapy were assessed in clinical trials, preclinical models were mostly used to evaluate the direct anticancer effects of CBD. This review focuses on the summary of studies published in recent years on the efficacy and mechanisms of action of CBD against four cancers: lung, breast, prostate, and colorectal cancer (Table 1). These four types of cancer were selected because they are the most common causes of cancer cases and deaths in the United States [19], as well as globally [25].

## 3. Efficacy and Mechanism of CBD on Cancer

### 3.1. Lung Cancer

One of the most frequently studied cancer types related to CBD treatment is lung cancer, which is the leading cause of cancer death in the United States [19]. The majority (~84%) of lung cancers are non-small cell lung cancer (NSCLC), and the rest are small cell lung cancer (SCLC; ~13%) [19]. In general, SCLC tends to grow and metastasize faster than NSCLC. In a study using lung cancer cell lines, including A549, H358, and H460 NSCLC, low doses of CBD (up to 3 µM) significantly attenuated intercellular adhesion molecule-1 (ICAM-1)-dependent cell invasion via cannabinoid receptors, TRPV1, and p42/44 mitogen-activated protein kinase (MAPK) [26]. CBD (3 µM) also inhibited ICAM-1-dependent cell invasion in two NSCLC-patient-derived primary lung tumor cell lines, and its efficacy was comparable to that of THC at the same concentration [26]. In a follow-up study using athymic nude mice xenografted with A549 cells, the injection of CBD (5 mg/kg) significantly decreased the size of xenografted tumors and the number of metastatic nodules in the lungs, with ICAM-1 and TIMP-1 as the key molecular targets of the anti-invasive mechanism of CBD [26]. In A549, H460 NSCLC cells, and metastatic cells derived from a lung cancer patient, CBD (3 µM) elevated the susceptibility of these lung cancer cells to lymphokine-activated killer (LAK) cell-mediated tumor-cell killing in an ICAM-1-dependent manner [30]. Ramer et al. conducted several other cell cultures (using A549, H460, and H358 cells, primary lung cancer cells) and animal-based (A549-xenografted nude mice) studies, in which they found that low doses of CBD (i.e., up to 10 µM in cells and 5 mg/kg in mice) suppressed cell growth and invasiveness, shrink tumor size, and decrease the number of metastatic nodules [27,28,29]. The anticancer effects of CBD were mainly attributed to the inhibition of plasminogen activator inhibitor-1 (PAI-1) and the activation of TIMP-1, p42/44 and p38 MAPKs, cyclooxygenase-2 (COX-2)-dependent prostaglandins, and PPAR-γ–dependent apoptotic cell death. These reports also suggested that CB1, CB2, and TRPV1 receptors played important roles in the anti-invasive activity of CBD [27,28,29].

Drug resistance continues to be a principal factor contributing to therapeutic failure in cancer patients. CBD was found to suppress the growth and metastasis of cisplatin-resistant NSCLC [33]. Misri et al. reported that CBD at higher concentrations (>10 µM) significantly reduced the viability of cisplatin-resistant NSCLC cells, stem-cell sphere formation, and stemness gene expression, accompanied by the activation of apoptosis and reactive oxygen species (ROS) [33]. They also found that CBD mediated its anticancer effects in part via an ion channel receptor, TRPV2, whose expression correlated with better overall survival for lung cancer patients [33]. In their follow-up study using NSG mice subcutaneously injected with cisplatin-resistant H460 cells, the injection of CBD (10 mg/kg) significantly diminished tumor progression and metastasis and suppressed cancer stem cell properties [33]. The upregulation of ICAM-1 was found in several types of cancer, such as breast and lung cancer—the major cancer killers. ICAM-1 plays a key role in tumor progression and prognosis, partly because it enhances the metastatic ability of malignant tumors [51,52]. TRPV is a member of the transient receptor potential (TRP) channels (which act as sensory mediators), which can be activated by endogenous ligands, heat, and mechanical and osmotic stress. Several TRP channels, including TRPV1 and TRPV2, are linked to cancer, especially at the later stages [53]. The deregulated expression and/or activity of these channels are associated with cancer progression via abnormal cell proliferation, differentiation, and death, resulting in the uncontrolled expansion of the transformed cells [53]. Thus, these studies suggest that CBD could be used as a potential therapeutic agent targeting ICAM-1 and TRPVs to attenuate the growth and metastasis of malignant tumors.

In addition to NSCLC, CBD was able to suppress the growth of SCLC. CBD (up to 48 µM) significantly reduced the cell viability of NSCLC (A549 and H1299) and SCLC (H69) cells [32]. Furthermore, CBD decreased the cancer stem cell spheres of both NSCLC and SCLC. When these three cell lines were treated with 10 µM of CBD, sphere formation and the expression of cancer stem cell genes (*SOX2*, *POU5F1*, *CD44*, or *PROM1*) were significantly decreased [32]. Moreover, CBD (10 µM) induced cell death by activating caspases 3 and 7, elevated the expression of pro-apoptotic genes (*TP53*, *CDKN1A*, *BAD*, *BCL2*, *BAX*, or *BAK1*) and the levels of ROS, and resulted in a loss of mitochondrial membrane potential in both cancers [32]. Apoptosis, a programmed cell-death process, is a target of anticancer therapy. Apoptosis is mainly regulated by the caspase and Bcl-2 families of proteins [54]. Among caspase proteins, executioner caspases 3 and 7 play a critical role in apoptosis. Once cleaved by initiator caspases, a series of hallmark features of apoptosis, including membrane blebbing, cell shrinkage, the formation of apoptotic bodies containing substances from dying cells, and the fragmentation of chromosomal DNA, are initiated and ultimately result in cell death [55]. Several anticancer agents approved by the United States Food and Drug Administration (FDA) either directly target apoptotic pathways in cancer cells or indirectly affect apoptosis-dependent cell survival and/or proliferation pathways [56]. Toxic levels of ROS in cells can induce apoptosis by activating pro-apoptotic effectors, such as Bcl-2, cytochrome c, and JNK pathways [56]. Thus, the anticancer activity of CBD can be partially attributed to its ability to induce ROS and, subsequently, apoptosis.

The suppressive effect of combinations of THC and CBD on lung cancer were also examined. In a study using A549, H460, and H1792 NSCLC cell lines, the in vitro assessment of the anti-proliferative effects of THC and CBD separately and in combination showed that THC (30 µM) and CBD (30 µM) significantly suppressed NSCLC cell proliferation through the inhibition of epithelial-to-mesenchymal transition (EMT) and epidermal growth factor (EGF)-induced cell migration [31]. The THC:CBD combination (10 µM each) was the most effective at inhibiting NSCLC cell proliferation and in downregulating the gene expression of epidermal growth factor receptor (EGFR), which was not observed in the treatments with cannabinoids alone [31]. EGFR and vascular endothelial growth factor (VEGF), two independent but interrelated signaling proteins, are critical in the growth and metastasis of tumors. Clinically, an array of anticancer drugs targeting these pathways, such as Bevacizumab, Sorafenib, Cetuximab, Erlotinib, Gefitinib, and Panitumumab, has been developed to treat cancer, either alone or in combination with chemotherapy [57]. Further research on the potential synergistic effect of CBD and other cannabinoids or anticancer treatments against lung cancer progression and its underlying mechanisms of action are warranted, as the enhanced efficacy may lower the required dosage for each agent in the combination, thereby leading to milder side effects [58,59].

### 3.2. Breast Cancer

Breast cancer is the most common cancer in women in the United States, excluding skin cancers [19]. Breast cancer that has estrogen receptors is estrogen receptor-positive type, while the triple-negative type lacks the expression of estrogen, progesterone, and human epidermal growth factor receptors that are commonly found in breast cancer cells. The triple-negative type is considered the most aggressive clinical form of breast cancer [60]. It accounts for 10–15% of all breast cancer cases [19]. Among all the cancer types, the inhibitory effects of CBD are most frequently studied in breast cancer models [61]. Because the effect and mechanisms of CBD in breast cancer have been well reviewed by Almeida et al. [61], in this section, we focus on the studies published in the last three years. Similar to the findings on lung cancer cells, the localization and expression of TRPV1 might play an important role in CBD’s anticancer activity on breast cancer cells. In a study using MCF7, an estrogen receptor-positive cell line, and MDA-MB-231, a triple-negative cell line, CBD (20 µM) selectively targeted ROS-induced endoplasmic reticulum stress and unfolded protein response (UPR) activation in MCF7, but not in MDA-MB-231 [34]. The elevated intracellular ROS in MCF7 cells was induced by Ca^2+^ influx through the TRPV1 receptor [34]. Even though CBD (20 µM) inhibited the growth of MDA-MB-231 cells, oxidative stress-induced endoplasmic reticulum stress and UPR activation were not involved. This may have been due to the different localization of the TRPV1 receptor in these two cell lines [34]; MCF7 cells express TRPV1 on the plasma membrane and in the cytosol, whilst MDA-MB-231 mainly express tit on the endoplasmic reticulum and Golgi apparatus [62]. Another study using MCF7 cells showed that CBD was able to trigger a decrease in bound NAD(P)H and an increase in the mitochondrial concentrations of ROS and Ca^2+^, accompanied by changes in mitochondrial morphology [35]. In estrogen receptor-positive breast cancer cells that overexpressed aromatase (MCF-7aro), CBD (up to 20 µM) greatly suppressed cell growth by disrupting cell cycle progression and inducing autophagy to promote apoptosis, accompanied by inhibited aromatase activity and estrogen receptor α expression and enhanced estrogen receptor β expression [36]. Notably, CBD (5–10 µM) exerted stronger effects on most of these endpoints than THC and endocannabinoid anandamide, suggesting CBD as a potential therapeutic agent for estrogen receptor-positive breast cancer [36].

In a study using four breast cancer cell lines (MCF7, MDA-MB-231, T47D, and SK-BR-3), CBD (up to 7 µM) diminished the angiogenesis (blood vessel formation) and stem cell-like properties of these breast cancer cells through the downregulation of hypoxia-induced factor-1α (HIF-1α) and Src/von Hippel–Lindau tumor suppressor protein (VHL) signaling [37]. Similar to the finding in NSCLC cells [31], a low dose of CBD (2 µM) was able to decrease the expression of the EMT-related proteins Slug and Vimentin in breast cancer cells [37]. In addition to regular 2D cell cultures, the efficacy and mechanism of CBD against breast cancer in 3D cultures was assessed by Surapaneni et al. [38]. Interestingly, the IC_50_ values of CBD in 3D cultures of triple-negative breast cancer MDA-MB-231 and MDA-MB-468 cells were much higher than in 2D cultures, reaching 20.18 and 33.85 µM, respectively (vs. 3.22 and 3.31 µM in the 2D cultures, respectively) [38]. 3D cell cultures generally mimic the in vivo settings of tumor microenvironments better than 2D cultures due to the complexity and heterogeneity of tumor microenvironments, such as cell–cell interactions, which could partially explain the higher IC_50_ of CBD in 3D cultures [38].

Chemotherapy is one of the most frequently applied cancer treatments. In recent years, the potential synergistic effects of CBD and chemotherapeutic drugs against breast cancer have also been evaluated [38,39,40]. Doxorubicin (DOX) is a chemotherapy drug that is commonly used to treat many types of cancer, such as breast cancer, bladder cancer, and lymphoma. However, it causes significant side effects, most notably cardiotoxicity, at high doses [63]. Two recent studies reported that when combined with CBD, DOX could be more effective than when administered alone [38,39]. When CBD (1–5 µM) was added to 0.5–1 µM of DOX, an increase in anti-proliferative and pro-apoptotic effects was observed. Furthermore, the protein expression of caspase-9, an initiating caspase of intrinsic apoptosis [64], was greatly increased with the combination treatment in MDA-MB-468 triple-negative breast cancer cells, and the effect was superior to either agent alone [38]. Their follow-up study fabricated extracellular vesicles encapsulated with CBD (CBD EVs) through sonication for sustained release and improved intracellular and intratumoral uptake [39]. Importantly, CBD EVs sensitized MDA-MB-231 xenograft tumors to DOX through the suppression of pro-inflammatory proteins and the activation of pro-apoptotic markers [39]. In addition to DOX, Alsherbiny et al. evaluated the effects of a combination of CBD with four more chemotherapeutic drugs, including docetaxel, paclitaxel, vinorelbine, and 7-ethyl-10-hydroxycamptothecin in MCF7 cells [40]. Enhanced effects were observed with the combination of CBD (38.42–64.6 µM) and all the chemotherapeutic drugs, while the strongest synergism was found between CBD, vinorelbine, and 7-ethyl-10-hydroxycamptothecin. A major molecular mechanism underlying the synergistic effects was the boosting of the pro-apoptotic activity of these chemotherapeutic drugs in MCF7 cells by the CBD [40]. The enhancement of anticancer efficacy by CBD may help to reduce the required dosage of these drugs in chemotherapy, which, in turn, may alleviate their adverse effects. Since triple-negative breast cancer cases typically have a poorer prognosis [60], CBD and/or its combination with chemotherapeutic drugs may offer additional treatment options for patients.

### 3.3. Prostate Cancer

Prostate cancer is the second leading cause of cancer death in men in the United States, behind lung cancer [19]. Compared to breast, lung, and colorectal cancer, the protective effect of CBD against prostate cancer was examined only in a small number of studies. The androgen receptor (AR) plays a complicated, yet vital, role in the normal function of the prostate, as well as in the progression of prostate cancer. AR-negative prostate cancer is generally linked with a more unfavorable prognosis than the AR-positive type [65]. A cell-culture study using the AR-positive prostate cancer cell line, LNCaP, suggested that CBD (up to 15 µM) was successful at initiating the apoptosis of cancer cells and acting as an antiproliferative agent [41]. Interestingly, the induction of apoptosis in LNCaP cells was dependent on phosphatase, but was independent of cannabinoid receptors [41]. Petrocellis et al. studied the effects of CBD on AR-positive (LNCaP and 22RV1) and AR-negative (DU-145 and PC-3) cells and found that CBD (1–10 µM) significantly diminished the viability of all four cell lines and the expression of AR in the LNCaP and 22RV1 cells [23]. Similar to the observations from many other studies, it was reported that the anti-proliferative effects of CBD occurred through the activation of apoptosis, accompanied by an increase in the markers of intrinsic apoptotic pathways (p53-upregulated modulator of apoptosis (PUMA), C/EBP homologous protein (CHOP), and intracellular Ca^2+^), p53 (in the LNCaP cells only), and ROS [23]. The cellular stress mediators, the p53 family, and their downstream transcriptional targets, such as PUMA and Noxa, play an important role during apoptosis. Cellular stress, including oxidative stress, DNA damage stress, and unfolded protein stress, typically lead to cell death and the clearance of stressed cells [66]. CHOP is involved in endoplasmic reticulum stress-induced apoptosis, in which the prolonged activation of unfolded endoplasmic reticulum proteins stimulates apoptotic cell death via the upregulation of CHOP [67].

In another study on AR-negative PC-3 cells, a new mode of action underlying the anticancer effect of CBD was reported. It was found that CBD (1 and 5 µM) inhibited the release of exosome and microvesicle (EMV) and modulated EMV biogenesis, accompanied by the reduced expression of exosomal marker CD63, prohibitin, and STAT3 in PC-3 cells [42]. EMV is released by most of the body’s cells and plays an important role in intercellular communication by transferring genetic material (DNA, mRNA, miRNA, etc.) and proteins. The increased release of EMV is associated with cancer through the delivery of the oncogenic factors to normal cells, thereby leading to cancerous transformation [68]. Thus, as an EMV-inhibiting agent, CBD may sensitize malignant tumors to chemotherapy and slow down cancer progression in vivo [68].

### 3.4. Colorectal Cancer (CRC)

CRC is the third most common cancer diagnosed in the United States [19]. The inhibitory effect of CBD against CRC has been well studied in the past decade. Similar to lung, breast, and prostate cancer, the suppression of cell proliferation and xenograft tumor growth, the induction of ROS and apoptosis, and the activation of CB and TRPV receptors have been observed as a result of treating CRC with CBD. In SW480 [41], Caco-2 [43], HCT116 [43,44,45], and DLD-1 [44] CRC cells, low doses of CBD (up to 15 µM) significantly reduced cell proliferation through the direct or indirect activation of CB1, CB2, TRPV1, PPARγ, and/or G protein-coupled receptor 55 (GPR55) receptors. However, these effects may be cell line- and dose-dependent. A common antiproliferative mechanism of CBD found across many CRC studies is its ability to regulate pro- and anti-apoptotic proteins and mediators, thereby inducing the cellular apoptosis of CRC cells and tumors. For example, CBD (6 µM) triggered the apoptosis of HCT116 and DLD-1 cells by regulating the expression of CHOP, inositol-requiring enzyme-1α (RE1α), and phosphorylated protein kinase RNA-like ER kinase (PERK) in a Noxa- and ROS-dependent manner [46]. The transmembrane proteins, RE1α and PERK, are both involved in the upstream regulatory pathways of CHOP. Unfolded endoplasmic reticulum proteins trigger the oligomerization and autophosphorylation of RE1α and PERK, ultimately leading to the upregulation of CHOP and the activation of apoptosis [67]. In HCT116, HT29, and DLD-1 cells, CBD (4 µM) modulated the expression of CHOP, PERK, and death receptor DR5, and induced TNF-related apoptosis-inducing ligand (TRAIL)-dependent apoptosis [47]. CHOP can stimulate apoptosis through pathways including mitochondria-dependent death receptors. Death receptor-mediated apoptosis is activated when death ligands, such as tumor necrosis factor (TNF) and TRAIL, bind to death receptors, such as DR4 and DR5 [67].

The activation of cellular apoptosis was also observed in animal studies with CDB intervention. In colon-specific carcinogen azoxymethane (AOM)-treated mice, CBD (1 mg/kg) diminished AOM-induced aberrant crypt foci (AFC), polyp, and tumor formation, and Akt activation, as well as stimulating principal pro-apoptotic protein caspase-3 [43]. Akt is also involved in CHOP-dependent apoptosis, acting as a direct modulator of caspases and mitochondrial pro-apoptotic proteins [69]. In BALB/c nude mice injected with HCT116 Luc+ cells (a luminescent cell line derived from HCT116), CBD (20 mg/kg) significantly suppressed the growth of xenograft tumors and elevated cellular apoptosis and Noxa expression in the tumor tissue [46]. Another study, using BALB/c mice injected with CT26 (a mouse CRC cell line), also found that the inhibition of xenograft tumors by CBD (1 and 5 mg/kg) was accompanied by the induction of apoptosis and oxidative stress parameters, including superoxide dismutase (SOD), glutathione peroxidase (GPx) and glutathione reductase (GR) activity, and total antioxidant capacity [49]. Interestingly, oxidative stress plays a biphasic role in carcinogenesis, partly depending on the concentration of ROS, with high levels of ROS often being cytotoxic [70]. ROS is a potent apoptosis stimulator that is capable of inducing the intrinsic mitochondrial pathway, the extrinsic death receptor, and the endoplasmic reticulum stress pathway of apoptosis by altering the permeability of the inner mitochondrial membrane and the activity of the mitochondrial permeability transition pore complex (MPTP), leading to the release of cytochrome c and the activation of caspases [70]. On the other hand, ROS can be pro-carcinogenic. Specifically, ROS is known to cause DNA damage, which is typically involved in the pathogenesis of cancer [70]. In Caco-2 and HCT116 cells, CBD (10 µM) protected cells against hydrogen peroxide-induced oxidative damage to DNA molecules [43]. The role of CBD in ROS generation may be contingent upon concentration, treatment time, and/or cell line, which warrants further investigation.

In addition to apoptosis, necrosis is another major mechanism of cell death. The major difference between these two cell death modes is that apoptosis is a highly regulated physiological process, whereas necrosis is considered pathological. Autophagy is a cytoprotective process that can be induced as an adaptive response by endoplasmic reticulum stress. It is linked to both apoptosis and necrosis, exerting a biphasic function that is either pro-survival or pro-death [71]. The crosstalk between apoptosis, necrosis, and autophagy is rather complex and beyond the scope of the current review. However, it is well recognized that the modulation of cell death and autophagy has important implications for cancer pathogenesis and treatment. In HT-29 cells, CBD (30 µM) attenuated cancer cell growth and induced necrosis, accompanied by diminished glutathione (GSH)-to-oxidized-glutathione (GSSG) ratio, level of ascorbic acid (AA), activity of catalase (CAT), and GPx and GR, as well as elevated levels of malondialdehyde (MDA) [50]. In this study, CBD induced oxidative stress and ROS production in HT-29 cells, which appeared to consume GSH and suppress the activity of the antioxidant enzymes CAT, GR, and GPx [50]. The effect of CBD on autophagy was examined in oxaliplatin-resistant DLD-1 and colo205 cells [48]. Although oxaliplatin is a standard chemotherapeutic drug for CRC treatment, patients often develop resistance to oxaliplatin, which leads to treatment failure. In oxaliplatin-resistant DLD-1 and colo205 cell lines, CBD (4 µM) induced autophagic cell death by decreasing nitric oxide synthase 3 (NOS3) activity and the activation of the AMP-activated protein kinase (AMPK), TOR, and Akt signaling pathways [48], all of which are key signaling proteins involved in tumorigenesis [72]. NOS3 mainly participates in the generation of nitric oxide (NO) and has been found to promote the proliferation, angiogenesis, and invasiveness, as well as suppress the apoptosis, of cancer cells [73]. ROS and the autophagic markers, LC3 and p62, were increased by CBD via antioxidant SOD2, causing mitochondrial dysfunction [48]. Altogether, CBD sensitizes CRC cells to oxaliplatin treatment, which may help alleviate drug resistance in chemotherapy.

Other mechanisms of CBD were also observed in CRC cells. For instance, CBD (1 and 2.5 µM) significantly prevented the adhesion of HCT116 cells to endothelial cells and suppressed their invasiveness and migration via the GPR55 receptor [45]. In BALB/c mice injected with CT26 murine CRC cells, CBD (1 and 5 mg/kg) exerted suppressive effects on tumor growth and cellular pleomorphism by diminishing the gene expression of VEGF, a key mediator of angiogenesis in cancer, and serum levels of the proinflammatory cytokines, IL-6 and IL-8 [49]. CBD’s inhibition of VEGF [49] and EGFR [31] might help explain its protective effect against tumor invasiveness, migration, and metastasis. The proinflammatory cytokines, IL-6 and IL-8, are key mediators of inflammation. It is known that chronic inflammation plays an essential role in all stages of tumorigenesis [74]. Specifically, poor prognosis in CRC is correlated with the presence of proinflammatory cytokines [74]. The anti-inflammatory effects of CBD have been demonstrated in a variety of experimental settings [75], but their implication in cancer treatment requires further research.

## 4. Discussion and Conclusions

Although CBD shows promising positive effects on cancer management in numerous preclinical studies and some human clinical trials, mechanistic studies on the efficacy of CBD’s anticancer effects are still limited. Emerging evidence suggests that CBD and other cannabinoid treatments may relieve cancer pain and ease the side effects of chemotherapy. However, research on CBD as a cancer treatment and its underlying mechanisms of action, especially in large clinical trials, are warranted. In addition to the limited knowledge on the anticancer efficacy, mechanisms of action, and potential side effects of CBD in cancer treatment, another major challenge in using CBD as a cancer therapy is that variations exist among CBD-based products. The quality of *Cannabis* cultivation and the CBD constituents of commercial products can vary significantly, as *Cannabis* contains more than 100 cannabinoids [17]. Synthetic, highly potent cannabinoid products that act as full cannabinoid receptor agonists are also available on the market; many of these have been banned by the U.S. government because they may cause severe illness, and even death [76,77]. Moreover, different routes of drug administration and habits of consumption may also substantially affect the pharmacokinetics of CBD, thereby altering its effectiveness. Similar to other medicinal plants, the standardization of the concentration and composition of CBD-based anticancer drugs is of great importance.

**Figure 1:**
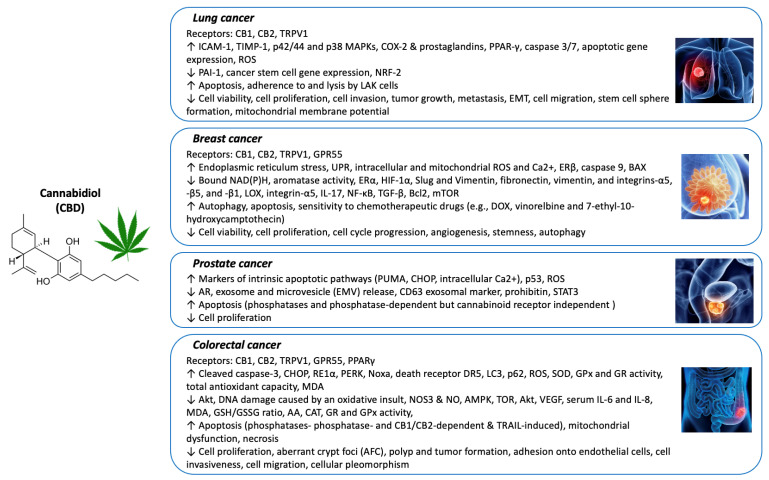
Major anticancer mechanisms of CBD on lung, breast, prostate, and colorectal cancers.

Through this review, we delineated the relationship between CBD treatment and its anticancer effects. Based on mostly cell culture-based studies and a few animal models, it is possible that complex and diverse molecular mechanisms are involved in the anticancer activity of CBD. Among the most commonly reported are the activation of the CB1, CB2, and TRPV1 receptors, the induction of apoptosis in cancer cells, the suppression of the invasiveness, migration, and metastasis of tumors, and the enhancement of the effectiveness of chemotherapeutic drugs (Figure 1). As CBD treatment for cancers has become more widely available in recent years, further research efforts are warranted to obtain knowledge about the impact of CBD on cancer. Overall, the current study supports the notion that CBD can offer positive outcomes in cancer treatment. Future research in the area of CBD and cancer should aim to examine the efficacy and safety of CBD in human clinical trials, especially in randomized controlled trials, as they are considered the gold standard for studying causal relationships.

## Figures and Tables

**Table 1:** Anticancer studies of CBD.

Cancer Type	Model	Dosage/ Treatment	Effects	Reference
Lung Cancer	Non-small cell lung cancer (NSCLC) A549, H358, and H460 cell lines, and human-derived NSCLC cells	Up to 3 µM CBD	In NSCLC cell lines: ↓ Intercellular adhesion molecule-1 (ICAM-1)-dependent cell invasion; ↑ ICAM-1 and matrix metalloproteinases-1 (TIMP-1) via cannabinoid receptors, transient receptor potential vanilloid 1 (TRPV1), and p42/44 mitogen-activated protein kinase (MAPK) In primary NSCLC cells: ↓ ICAM-1-dependent cell invasion; ↑ ICAM-1 and TIMP-1 expression, CBD showed comparable anti-invasive efficacy to THC (3 µM)	[26]
	Athymic nude mice xenografted with A549	5 mg/kg CBD by intraperitoneal injection	↓ Tumor size and number of metastatic nodules; ↑ ICAM-1 and TIMP-1 expression	
	A549, H460, and H358	Up to 1 µM CBD	↓ Cell invasion, plasminogen activator inhibitor-1 (PAI-1) via CB1, CB2, and TRPV1 receptors	[27]
	Athymic nude mice xenografted with A549	5 mg/kg CBD by intraperitoneal injection	↓ Tumor size, PAI-1 protein expression	
	A549	10 µM CBD	↓ Cell invasion; ↑ TIMP-1, p42/44, and p38 MAPKs via CB1, CB2, and TRPV1 receptors	[28]
	A549-xenografted nude mice	5 mg/kg CBD by intraperitoneal injection	↓ Number of metastatic nodules	
	A549 and H460, and primary cells from a lung cancer patient	3 µM CBD	↓ Cell viability; ↑ apoptosis, cyclooxygenase-2 (COX-2) and PPAR-γ, COX-2-dependent prostaglandins, PPAR-γ–dependent apoptotic cell death	[29]
	A549-xenografted nude mice	5 mg/kg CBD by intraperitoneal injection	↓ Tumor size and CD31 (vascularization marker); ↑ COX-2 and PPAR-γ	
	NSCLC A549 and H460 cell lines and human derived metastatic lung cancer cells	3 µM CBD	↑ Adherence to and lysis by lymphokine-activated killer (LAK) cells, ICAM-1 expression	[30]
	NSCLC cell lines A549, H460, H1792	Tetrahydrocannabinol (THC) 30 µm, cannabidiol (CBD) 30 µm, and combination THC:CBD 10 µm each	All treatments: ↓ cancer cell proliferation, epithelial-to-mesenchymal transition (EMT), epidermal growth factor (EGF)-induced cell migration THC:CBD combination: ↓ epidermal growth factor receptor (EGFR) gene	[31]
	A549 and H1299 NSCLC cell lines and H69 small cell lung cancer (SCLC) cell line	Up to 48 µM CBD; 10 µM CBD for treatment of stem cell spheres	↓ Cell viability, stem cell sphere formation, expression of cancer stem cell genes (*SOX2*, *POU5F1*, *CD44*, or *PROM1*), mitochondrial membrane potential; ↑ cell death, caspase 3/7 protein, expression of apoptotic genes (*TP53*, *CDKN1A*, *BAD*, *BCL2*, *BAX*, or *BAK1*), levels of reactive oxygen species (ROS)	[32]
	Cisplatin-resistant (CR) NSCLC cell lines H460 and A549	Up to 90 µM CBD	↓ Cell viability, nuclear factor erythroid 2-related factor 2 (NRF-2) expression; ↑ apoptosis, ROS, sphere formation and protein expression of Snail, Nanog, and Vimentin	[33]
	NSG mice injected with H460-CR cells	10 mg/kg CBD by intraperitoneal injection	↓ Tumor progression and metastasis	
Breast Cancer	MCF7 (estrogen receptor-positive) and MDA-MB-231 (triple-negative)	20 µM CBD	↓ Cell viability of both MCF7 and MDA-MB- 231; ↑ Endoplasmic reticulum stress, unfolded protein response (UPR) activation, intracellular ROS and Ca^2+^ accumulation via the activated TRPV1 receptor in the MCF7	[34]
	MCF7	Up to 20 µM CBD	↓ Bound NAD(P)H; ↑ mitochondrial concentrations of ROS and Ca^2+^	[35]
	Estrogen receptor-positive (ER+) aromatase-overexpressing MCF-7aro	Up to 20 µM CBD	↓ Cell viability, aromatase activity, ERα levels, cell cycle progression; ↑ autophagy, apoptosis, ERβ levels	[36]
	MCF7, MDA-MB-231, T47D, and SK-BR-3	Up to 7 µM CBD	↓ Cell viability, angiogenesis, stemness, hypoxia-induced factor-1α (HIF-1α) expression through Src/von Hippel–Lindau tumor suppressor protein (VHL) signaling, Slug and Vimentin (EMT-related proteins)	[37]
	MDA-MB-231 and MDA-MB-468 (triple-negative)	Up to 5 µM CBD in 2D cultures and up to 50 µM in 3D cultures	↓ Cell viability (CBD had greater IC50 values in 3D than 2D), fibronectin, vimentin, and integrins-α5, -β5, and -β1, autophagy	[38]
	MDA-MB-468	Up to 5 µM CBD in combination with doxorubicin (DOX)	↑ DOX sensitivity in cancer cells, caspase 9; ↓ LOX and integrin-α5	
	MDA-MB-231 cells and female nude mice injected with MDA-MB-468 cells	CBD-loaded extracellular vesicles (5 mg/kg)	↑ DOX sensitivity in cancer cells and xenograft tumors, caspase 9, and BAX; ↓ interleukin-17 (IL-17), NF-κB, TGF-β, Bcl2 and mTOR	[39]
	MCF7	38.42–64.6 µM CBD in combination with DOX, docetaxel, paclitaxel, vinorelbine, and 7-ethyl-10-hydroxycamptothecin	Enhanced effects were observed with the combination of CBD and all chemotherapeutic drugs, while the strongest synergism was found between CBD and vinorelbine and 7-ethyl-10-hydroxycamptothecin; ↑ apoptosis	[40]
Prostate Cancer	Androgen receptor (AR)-positive prostate cancer cell line LNCaP	Up to 15 µM CBD	↓ Cell proliferation; ↑ phosphatases and phosphatase-dependent apoptosis, but cannabinoid receptor independent	[41]
	AR-positive (LNCaP and 22RV1) and AR-negative (DU-145 and PC-3) cells	1–10 µM CBD	↓ Cell viability and AR (in LNCaP and 22RV1 cells); ↑ apoptosis, markers of intrinsic apoptotic pathways (p53-up-regulated modulator of apoptosis (PUMA), C/EBP homologous protein (CHOP) and intracellular Ca2+) partly due to TRPM8 antagonism, p53 (in LNCaP cells), and ROS	[23]
	PC-3	1 and 5 µM CBD	↓ Exosome and microvesicle (EMV) release, CD63 exosomal marker, prohibitin, and STAT3	[42]
Colorectal Cancer (CRC)	SW480	Up to 15 µM CBD	↓ Cell proliferation; ↑ phosphatases and phosphatase-, CB1/CB2-dependent apoptosis	[41]
	Caco-2 and HCT116	10 µM CBD	↓ Cell proliferation via CB1, TRPV1, and PPARγ receptors, Akt activation, and DNA damage caused by an oxidative insult	[43]
	CRC induced by azoxymethane (AOM) in male ICR mice	1 and 5 mg/kg CBD by intraperitoneal injection	1 mg/kg: ↓ AOM-induced aberrant crypt foci (AFC), polyp and tumor formation, and Akt activation; ↑ apoptoic protein cleaved caspase-3 5 mg/kg: ↓ AOM-induced polyp formation	
	DLD-1 and HCT116	Up to 5 µM CBD	↓ Cell proliferation via CB1 receptor	[44]
	HCT116	1 and 2.5 µM CBD	↓ Adhesion of HCT116 cells onto endothelial cells, invasiveness, migration via G protein-coupled receptor 55 (GPR55)	[45]
	HCT116 and DLD-1	6 µM CBD	↓ Cell viability; ↑ apoptosis by regulating pro- and anti-apoptotic proteins (CHOP, inositol requiring enzyme-1α (RE1α), phosphorylated protein kinase RNA-like ER kinase (PERK), etc.), in a Noxa-and-ROS-dependent manner	[46]
	BALB/c nude mice injected with HCT116 Luc+ cells (a luminescent cell line derived from HCT116)	10 and 20 mg/kg CBD by intraperitoneal injection	20 mg/kg ↓ tumor size; ↑ apoptosis and Noxa expression	
	HCT116, HT29, and DLD-1	4 µM CBD	↓ Cell viability; ↑ apoptosis by regulating pro- and anti-apoptotic proteins (CHOP, PERK, death receptor DR5 expression by ER stress, etc.), TNF-related apoptosis-inducing ligand (TRAIL)-induced apoptosis	[47]
	Oxaliplatin-resistant DLD-1 and colo205	4 µM CBD	↓ Cell proliferation, nitric oxide synthase 3 (NOS3), nitric oxide (NO), AMP-activated protein kinase (AMPK), TOR, and Akt; ↑ autophagic markers LC3 and p62, ROS via superoxide dismutase 2 (SOD2) causing mitochondrial dysfunction	[48]
	BALB/c mice injected with CT26 (mouse CRC cells)	1 and 5 mg/kg CBD by intraperitoneal injection	↓ Tumor size, cellular pleomorphism, vascular endothelial growth factor (VEGF), serum levels of IL-6 and IL-8 (5 mg/kg), and malondialdehyde (MDA); ↑ apoptosis, SOD (5 mg/kg), glutathione peroxidase (GPx) and glutathione reductase (GR) activity, and total antioxidant capacity	[49]
	HT-29	30 µM CBD	↓ Cell viability, glutathione (GSH)-to-oxidized-glutathione (GSSG) ratio, ascorbic acid (AA), catalase (CAT), and GR and GPx activity; ↑ MDA and necrosis	[50]

## Data Availability

Not applicable.

## Abbreviations

cannabidiol	(CBD)
∆9-tetrahydrocannabinol	(THC)
cannabinoid receptor	(CB)
transient receptor potential	(TRP)
non-small cell lung cancer	(NSCLC)
Small cell lung cancer	(SCLC)
mitogen-activated protein kinase	(MAPK)
lymphokine-activated killer	(LAK)
cyclooxygenase-2	(COX-2)
reactive oxygen species	(ROS)
epithelial-to-mesenchymal transition	(EMT)
epidermal growth factor	(EGF)
epidermal growth factor receptor	(EGFR)
vascular endothelial growth factor	(VEGF)
unfolded protein response	(UPR)
hypoxia-induced factor-1α	(HIF-1α)
doxorubicin	(DOX)
Src/von Hippel–Lindau tumor suppressor protein	(VHL)
extracellular vesicles encapsulated with CBD	(CBD EVs)
androgen receptor	(AR)
p53-up-regulated modulator of apoptosis	(PUMA)
C/EBP homologous protein	(CHOP)
exosome and microvesicle	(EMV)
colorectal cancer	(CRC)
G protein coupled receptor 55	(GPR55)
inositol requiring enzyme-1α	(RE1α)
phosphorylated protein kinase RNA-like ER kinase	(PERK)
TNF-related apoptosis-inducing ligand	(TRAIL)
azoxymethane	(AOM)
aberrant crypt foci	(AFC)
superoxide dismutase	(SOD)
glutathione peroxidase	(GPx)
glutathione reductase	(GR)
mitochondrial permeability transition pore complex	(MPTP)
glutathione	(GSH)
oxidized glutathione	(GSSG)
ascorbic acid	(AA)
catalase	(CAT)
malondialdehyde	(MDA)
nitric oxide synthase 3	(NOS3)
AMP-activated protein kinase	(AMPK)
nitric oxide	(NO)
randomized controlled trials	(RCT)
